# ProtT5-MSCRNet: a multi-scale convolutional and channel-recalibrated deep learning framework for anticancer peptide prediction

**DOI:** 10.3389/frai.2026.1856815

**Published:** 2026-06-16

**Authors:** Zhi Li, Zhen Liu, Jianguo Zhong, Yuqin Jiang

**Affiliations:** 1 The First People’s Hospital of Jiashan, Jiashan Hospital Affiliated of Jiaxing University, Jiashan, China; 2 Department of Rehabilitation Medicine, The Second Affiliated Hospital of Chengdu Medical College (Nuclear Industry 416 Hospital), Chengdu, China; 3 Faculty of Humanities and Social Sciences, Macao Polytechnic University, Macao, China

**Keywords:** anticancer peptides, channel-wise attention, deep learning framework, multi-scale feature learning, ProtT5

## Abstract

**Introduction:**

Cancer is a leading cause of mortality worldwide. Anticancer peptides (ACPs) are promising therapeutic candidates due to their low toxicity, favorable biocompatibility, and selective anticancer activity; however, experimental ACP identification and screening remain labor-intensive, time-consuming, and costly.

**Methods:**

We developed ProtT5-MSCRNet, an end-to-end deep learning framework for ACP prediction that integrates ProtT5-based evolutionary representations, multi-scale convolutional feature extraction, channel-wise attention recalibration, and robust optimization strategies.

**Results:**

Experiments on two independent benchmark datasets showed that ProtT5-MSCRNet outperformed state-of-the-art ACP prediction methods. The model achieved ACC/SN/SP/MCC values of 0.954/0.874/0.983/0.881 on Test Set 1 and 0.984/0.980/0.987/0.967 on Test Set 2. Ablation studies and visualization analyses further supported the effectiveness and interpretability of the proposed model.

**Discussion:**

ProtT5-MSCRNet provides a robust, accurate, and biologically meaningful computational tool for facilitating ACP identification and accelerating anticancer peptide discovery.

## 1 Introduction

Cancer has become one of the leading diseases threatening human health and survival worldwide, imposing a substantial burden on public health systems due to its high incidence and mortality rates (Eghtedari et al., 2021; Chhikara and Parang, 2023; Duan et al., 2024; Park et al., 2022). Although conventional treatment modalities, such as surgery, radiotherapy, and chemotherapy, continue to play important roles in cancer management, their therapeutic efficacy is often limited by severe side effects, drug resistance, and nonspecific damage to normal cells, which collectively compromise treatment outcomes and patients’ quality of life. Consequently, the development of effective, low-toxicity, and highly selective anticancer therapeutic strategies has emerged as a major focus in contemporary cancer research (Han et al., 2024; Zhang et al., 2021).

Anticancer peptides (ACPs) are a class of short peptides typically consisting of 5–50 amino acids and have attracted considerable attention owing to their favorable biocompatibility, relatively low toxicity, and selective interactions with cancer cell membranes (Deng et al., 2023; Ghaly et al., 2023; Marqus et al., 2017; Okeke et al., 2024). Accumulating evidence indicates that ACPs exert anticancer activity through multiple mechanisms, including disruption of cancer cell membrane integrity, induction of apoptosis, inhibition of tumor angiogenesis, and modulation of immune responses. Compared with conventional small-molecule chemotherapeutic agents, ACPs are less prone to inducing drug resistance and generally cause less damage to normal cells, highlighting their promising potential for clinical applications. Despite these advantages, experimental identification and screening of ACPs remain labor-intensive, time-consuming, and costly, with relatively low success rates (Wu et al., 2014; Ao et al., 2022). With the rapid advancement of bioinformatics and artificial intelligence technologies, computational approaches for ACP prediction have emerged as efficient and cost-effective alternatives (Ferdous et al., 2026; Ferdous et al., 2026). By modeling physicochemical properties, amino acid composition, sequence patterns, and structural characteristics of peptide sequences, machine learning and deep learning methods can effectively discriminate ACPs from non-ACPs, thereby substantially improving the efficiency of candidate peptide discovery.

In studies on anticancer peptide prediction based on manual feature engineering, substantial progress has been achieved over the past decade. Schaduangrat et al. (2019) developed a bioinformatics prediction tool named ACPred, which integrates traditional machine learning classifiers, including support vector machines (SVMs) and random forests, with diverse categories of peptide sequence features. This approach enabled accurate identification of anticancer peptides, achieving a cross-validation accuracy of 95.61%, while also providing a certain degree of interpretability with respect to underlying mechanisms of action. Subsequently, Huang et al. (2021) proposed a two-stage machine learning framework that combines sequence-derived features with physicochemical property descriptors. This method not only effectively discriminates ACPs from non-ACPs but also enables precise classification of functional ACP subtypes. Their study systematically analyzed amino acid composition at the N- and C-termini as well as the distribution of charged residues, yielding an overall accuracy of 88.99% and a Matthews correlation coefficient (MCC) of 0.75 on an independent test set. To address limitations related to dataset size and feature representation in existing ACP prediction tools, Sangaraju et al. (2024) constructed a comprehensively updated, high-quality benchmark dataset and, for the first time, incorporated spatial and probabilistic features into ACP representation. Based on a stacked deep learning architecture, they proposed mACPpred 2.0, which demonstrated significantly improved predictive performance compared with its predecessor and several widely used state-of-the-art tools. Furthermore, Liang et al. (2021) conducted a systematic evaluation of 16 mainstream ACP prediction tools using a unified benchmark dataset, thoroughly analyzing their performance characteristics and limitations. Building upon this comparative study, they introduced a novel ensemble-based framework termed ACPredStackL, which employs a stacking strategy to integrate multiple predictors. Experimental results indicated that this method exhibits competitive performance in anticancer peptide identification tasks.

With the rapid development of deep learning techniques (Cui et al., 2021; Xiao et al., 2024; Yan et al., 2024) for sequence modeling, researchers have increasingly applied these methods to anticancer peptide (ACP) prediction to reduce reliance on handcrafted features and improve representation learning. Ahmed et al. (2021) proposed ACP-MHCNN, a multi-head deep convolutional neural network that integrates discriminative features from peptide sequences, physicochemical properties, and evolutionary information, achieving notable performance improvements over previous methods. Han et al. (2022) introduced ACPred-BMF, a BiLSTM-based model with an attention mechanism that combines quantitative and qualitative amino acid features and binary profile encoding, achieving strong predictive performance while offering improved interpretability through feature visualization and Shapley value analysis. Subsequent studies further explored convolutional architectures. Ghulam et al. (2022) developed ACP-2DCNN, which constructs two-dimensional feature representations based on dipeptide statistics and applies 2D convolutional neural networks to enhance ACP classification accuracy. Yang et al. (2023) proposed CACPP, a framework that integrates TextCNN with contrastive learning to extract more discriminative high-level semantic features, consistently outperforming existing state-of-the-art methods and providing insights into sequence–function relationships through visualization analyses. More recently, pretrained protein language models have introduced new directions for ACP prediction. Wang et al. (2024) proposed iACP-DFSRA, which combines ProtBert embeddings with handcrafted features in a dual-channel architecture and employs attention mechanisms for feature fusion, achieving over 90% accuracy and strong MCC performance on independent test sets. Gao et al. (2025) further advanced this line of research with ACP-ESM2, a deep learning framework based on the ESM2 pretrained model and convolutional neural networks, which effectively captures evolutionary and local sequence patterns and achieves up to 97.6% accuracy, demonstrating robust and highly competitive performance in ACP identification. Zhou et al. (2026) proposed Aegis, a transformer-based framework that combines sequence-derived feature extraction, feature importance analysis, and optimal feature selection to achieve strong predictive performance, while also showing that ACP sequences are enriched in positively charged and hydrophobic residues. To provide a clearer overview of the development of computational ACP prediction methods, representative studies are summarized in Supplementary Table S1 in chronological order.

Despite this progress, several important limitations remain. First, some existing methods still depend heavily on handcrafted descriptors or shallow sequence encodings, which may not adequately capture the complex evolutionary and contextual information embedded in peptide sequences. Second, even when pretrained protein language models are adopted, they are often used only as static feature extractors, while the downstream architecture may lack sufficient capability to capture local structural patterns at different scales and to suppress redundant information in high-dimensional embeddings. Third, because available ACP datasets are relatively limited in size, models trained on rich embeddings are still vulnerable to overfitting and reduced generalization on independent test sets. Finally, ACP prediction is a task in which false negatives may be particularly undesirable, yet many existing studies still rely on conventional optimization objectives that do not explicitly emphasize hard or clinically valuable positive samples. These limitations indicate clear room for improvement in both feature modeling and training strategy.

**Figure 1:**
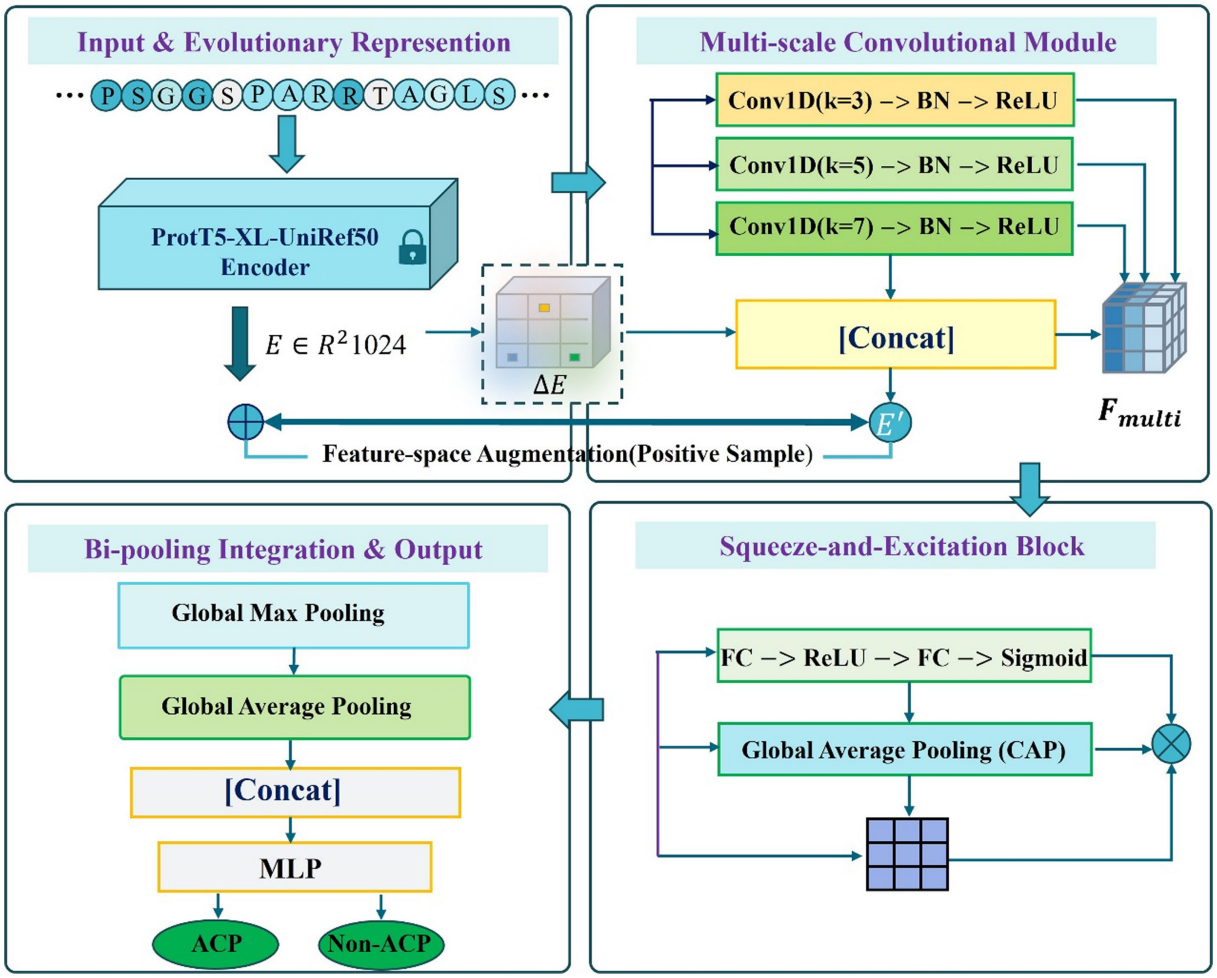
The overall architecture of the proposed ProtT5-MSCRNet framework.

To address the aforementioned limitations, we propose a novel end-to-end framework for anticancer peptide (ACP) prediction, termed ProtT5-MSCRNet (Multi-Scale Convolution and Recalibration Network). As illustrated in Figure 1, the proposed architecture integrates evolutionary representation learning, multi-scale feature extraction, channel-wise attention recalibration, and robust optimization strategies. Specifically, ProtT5 (Hao and Fan, 2024) is employed as the evolutionary representation encoder to generate high-dimensional embeddings enriched with contextual and biological semantics from peptide sequences. Based on these embeddings, a multi-scale one-dimensional convolutional module is designed to capture local structural patterns and functional motifs at different receptive fields. A Squeeze-and-Excitation (SE) channel attention mechanism is further introduced to adaptively recalibrate feature responses, highlighting informative channels while suppressing redundant signals. In addition, a perturbation-based feature-space augmentation strategy is adopted to improve robustness under limited-data conditions, and focal loss is used to facilitate more effective learning on informative and challenging samples in imbalanced classification settings. The overall workflow of the proposed method is as follows. First, peptide sequences are encoded into contextual representations using ProtT5. Next, multi-scale convolutional branches extract discriminative local patterns, and SE-based channel attention refines the resulting feature maps. The refined features are then integrated through bi-pooling and fed into the classifier for ACP prediction. Overall, ProtT5-MSCRNet enables accurate, stable, and biologically meaningful identification of ACPs.

The main novelty of this study lies not in the construction of a new dataset, but in the methodological design of the proposed framework. Specifically, the model integrates protein language model embeddings, local motif-aware representations, and handcrafted descriptors to capture complementary information at multiple representational levels. Moreover, the effectiveness of the proposed interaction-fusion strategy and biologically motivated local modeling is further supported by ablation experiments and local-window analysis, which demonstrate their direct contributions to improved predictive performance. The remainder of this paper is organized as follows. Section 2 describes the datasets, model architecture, and training strategy. Section 3 presents the experimental results, including comparisons with existing methods, embedding analysis, ablation studies, and interpretability evaluation. Section 4 concludes the study and discusses potential future directions.

## 2 Methods and materials

### 2.1 Overall workflow of the proposed technique

**Figure 2:**
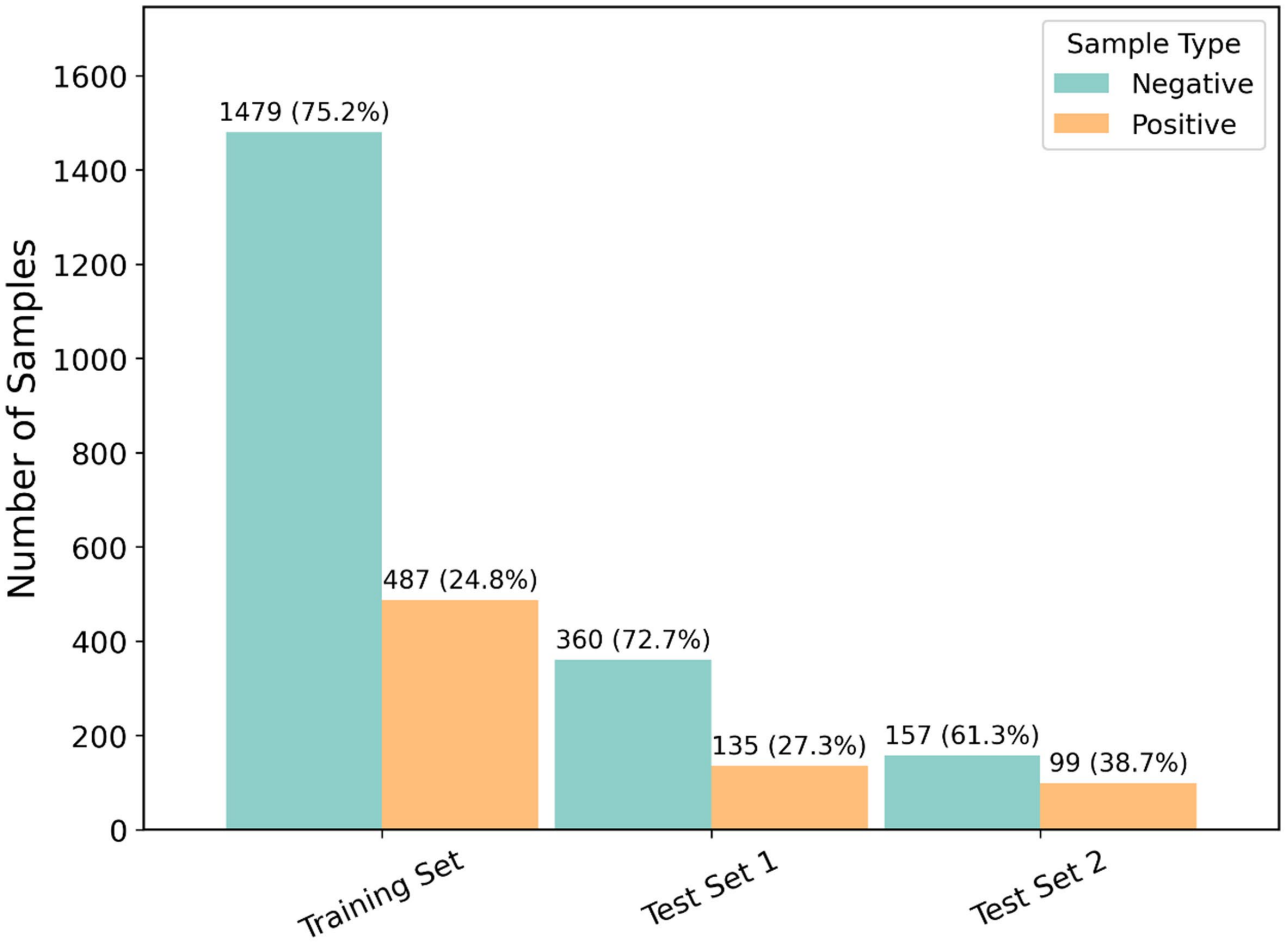
Distribution of ACP and non-ACP samples across the training and independent test datasets.

**Figure 3:**
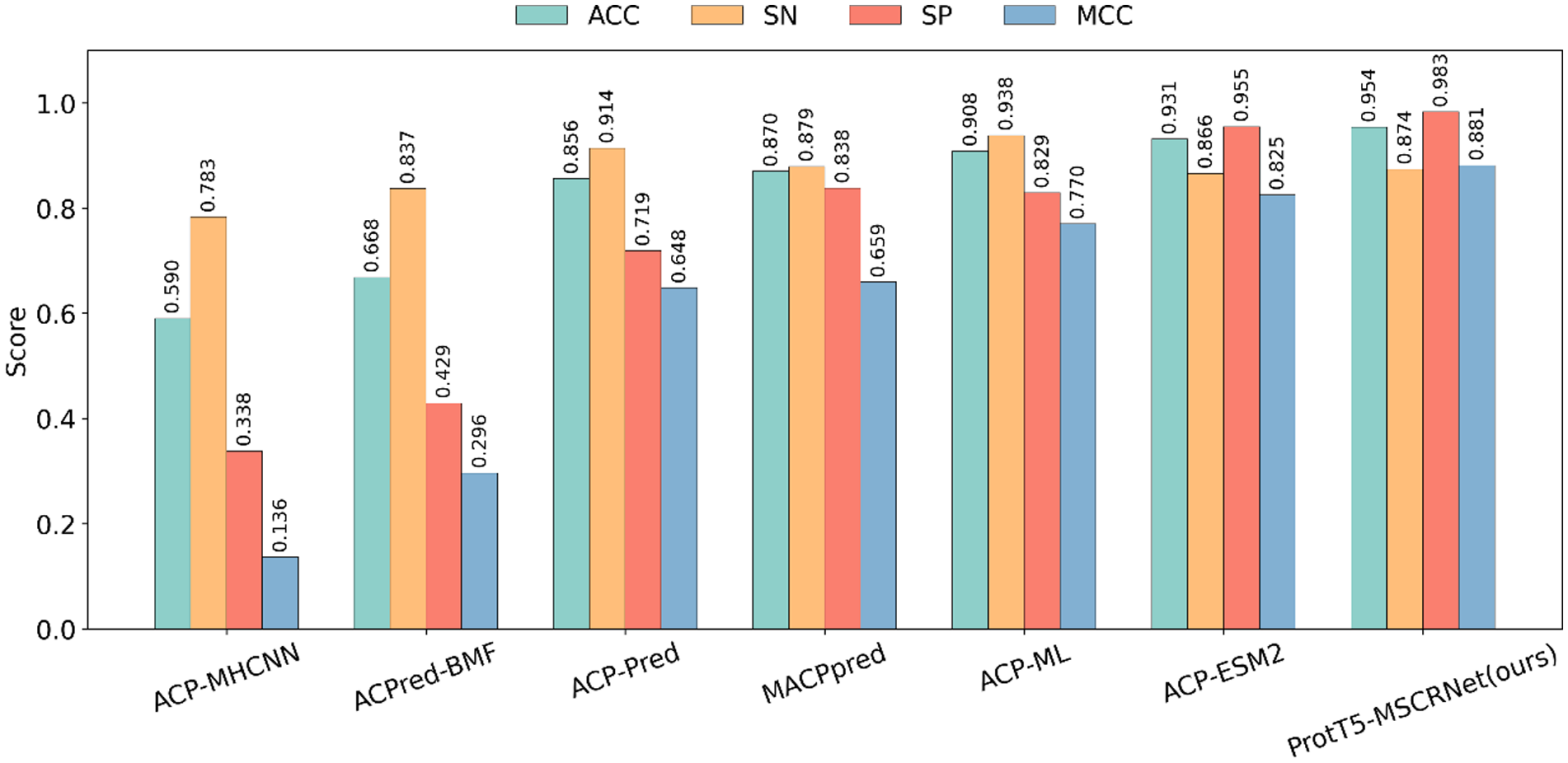
Performance comparison of ProtT5-MSCRNet and competing methods on Test Set 1.

**Figure 4:**
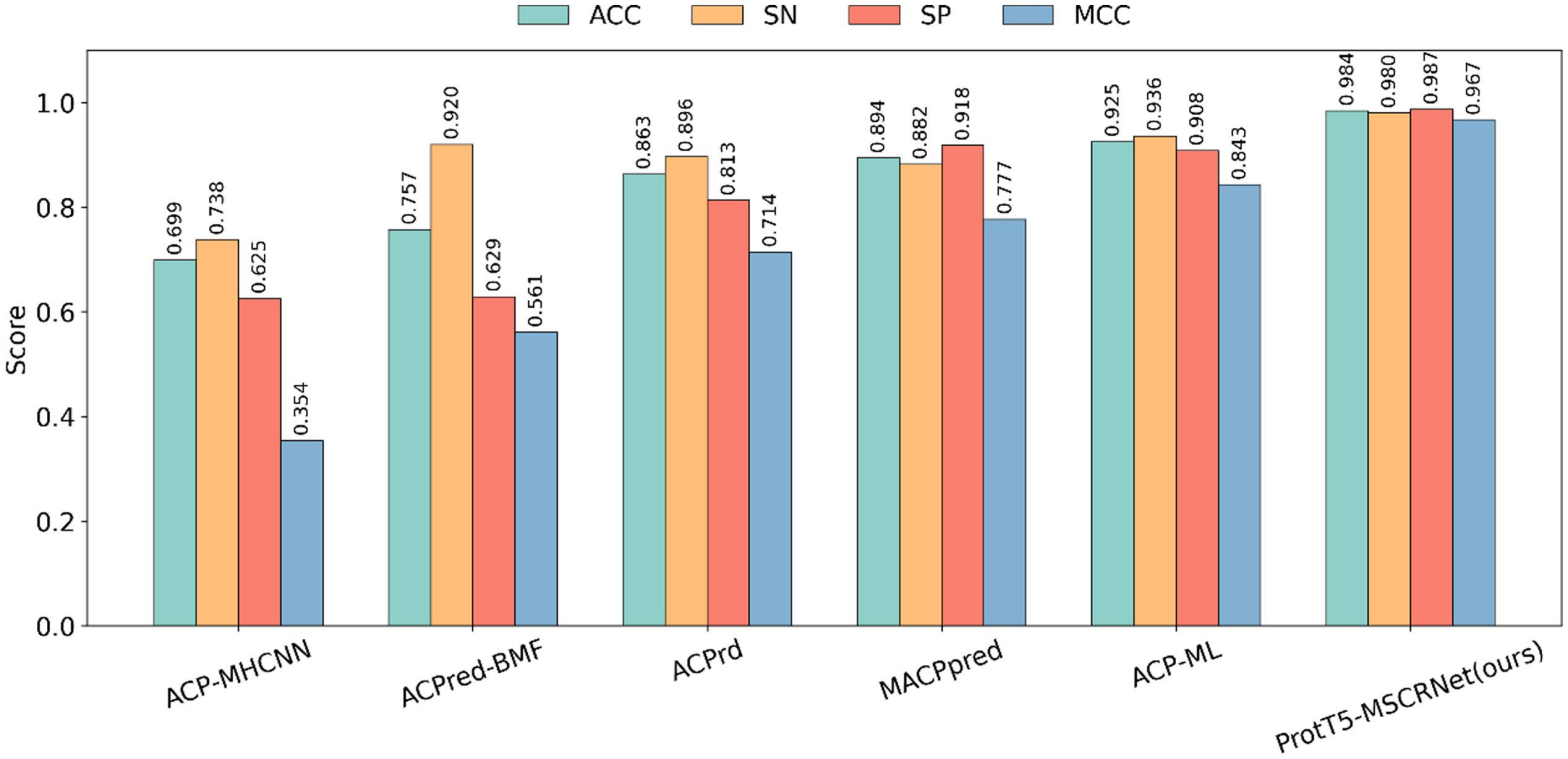
Performance comparison of ProtT5-MSCRNet and competing methods on the independent Test Set 2.

**Figure 5:**
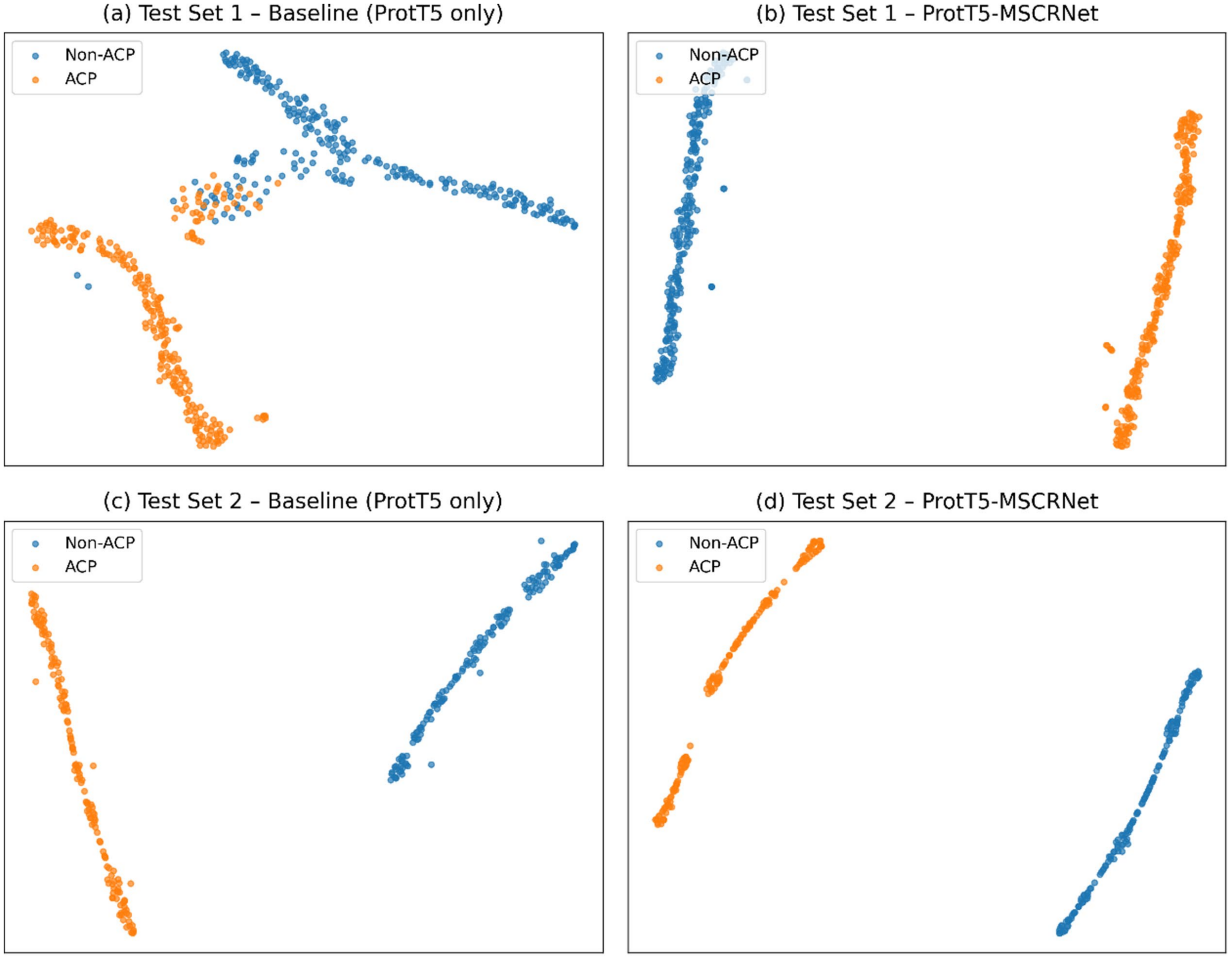
t-SNE visualization of feature representations learned by the baseline model and ProtT5-MSCRNet on Test Set 1 and Test Set 2. **(a)** Test Set 1 - Baseline (ProtT5 only). **(b)** Test Set 1 - ProtT5-MSCRNet. **(c)** Test Set 2 - Baseline (ProtT5 only). **(d)** Test Set 2 - ProtT5-MSCRNet.

**Figure 6:**
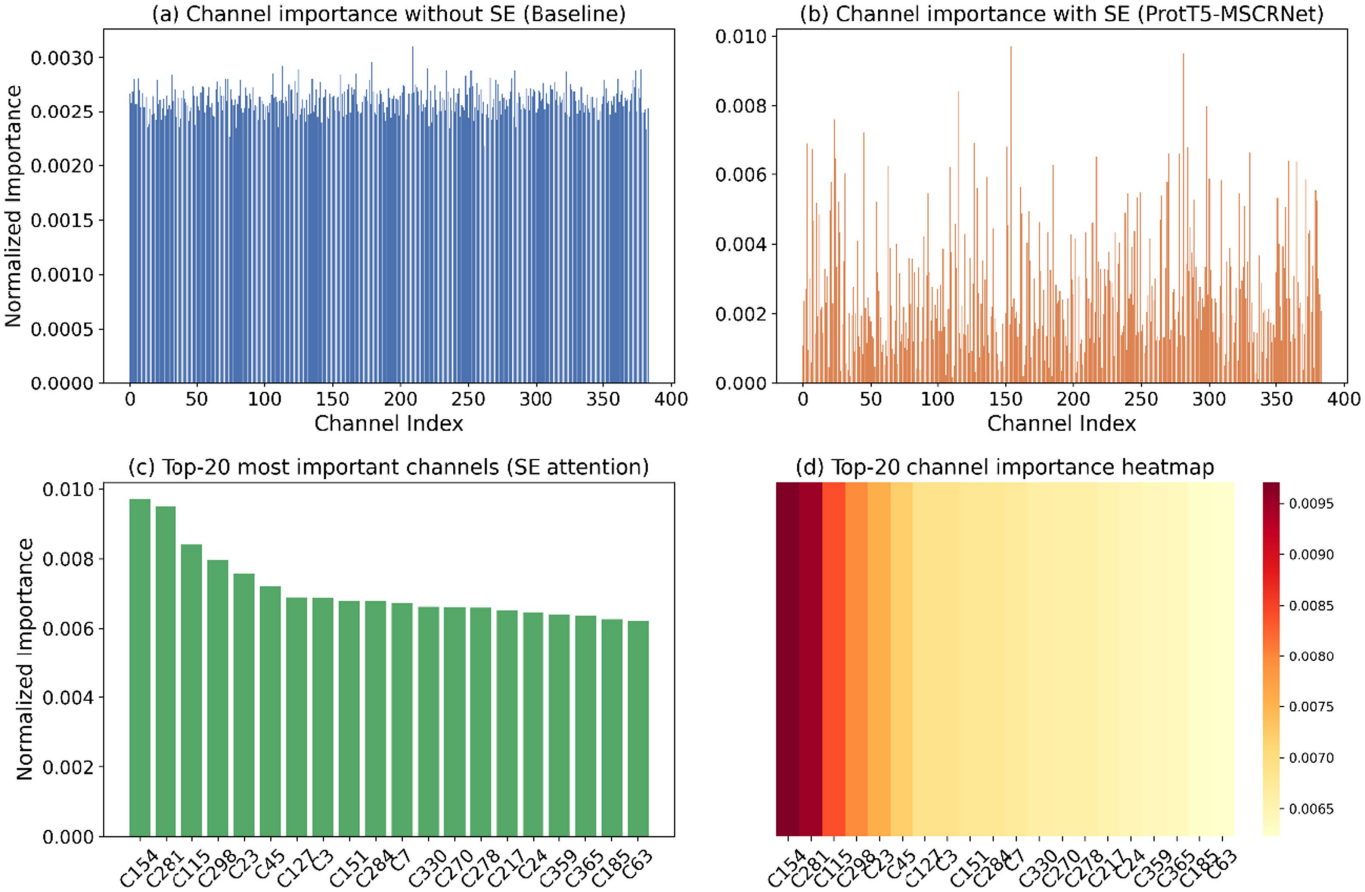
Channel importance analysis and visualization with and without SE attention in ProtT5-MSCRNet. **(a)** Channel importance without SE (Baseline). **(b)** Channel importance with SE (ProtT5-MSCRNet). **(c)** Top-20 most important channels (SE attention). **(d)** Top-20 channel importance heatmap.

To facilitate readability, the complete process of the proposed technique is summarized here in a step-by-step manner together with its corresponding visual elements. As illustrated in Figure 1, ProtT5-MSCRNet starts from raw peptide sequence input and follows an end-to-end predictive pipeline. The dataset composition and partitioning strategy are presented in Figure 2. After sequence input, ProtT5 is used to generate residue-level embeddings, which are then processed by the multi-scale convolution and SE-based recalibration modules for hierarchical feature learning. During training, feature-space augmentation and focal-loss-based optimization are applied to improve generalization and sensitivity. The predictive effectiveness of the resulting model is then evaluated by comparison with existing methods (Figures 3, 4), bootstrap-based confidence interval analysis (Table 1), embedding comparison experiments (Tables 2, 3), and ablation studies (Tables 4, 5). Finally, the interpretability of the learned representations is examined using t-SNE visualization and channel-wise importance analysis, as shown in Figures 5, 6, respectively.

**Table 1:** Bootstrap-based 95% confidence intervals of ProtT5-MSCRNet on the two independent test sets.

Test Set	Metric	Point estimate	95% CI
Test Set 1	ACC	0.954	0.935–0.971
Test Set 1	SN	0.874	0.817–0.927
Test Set 1	SP	0.983	0.969–0.994
Test Set 1	MCC	0.881	0.832–0.924
Test Set 2	ACC	0.984	0.968–0.996
Test Set 2	SN	0.980	0.949–1.000
Test Set 2	SP	0.987	0.969–1.000
Test Set 2	MCC	0.967	0.937–0.991

**Table 2:** Performance comparison of different protein embedding methods on Test Set 1.

Embedding method	ACC	SN	SP	MCC
ProtBERT	0.902	0.861	0.928	0.769
ESM2	0.931	0.866	0.955	0.825
ProtT5 (ours)	**0.954**	**0.874**	**0.983**	**0.881**

Bold values indicate the best performance among the compared embedding method.

**Table 3:** Performance comparison of different protein embedding methods on Test Set 2.

Embedding method	ACC	SN	SP	MCC
ProtBERT	0.947	0.952	0.939	0.893
ESM2	0.958	0.964	0.952	0.902
ProtT5 (ours)	**0.984**	**0.980**	**0.987**	**0.967**

Bold values indicate the best performance among the compared embedding method.

**Table 4:** Ablation results of ProtT5-MSCRNet on Test Set 1.

ProtT5	MSC	SE	Aug	Focal	ACC	SN	SP	MCC
√	×	×	×	×	0.921	0.862	0.958	0.812
√	√	×	×	×	0.934	0.868	0.971	0.836
√	√	√	×	×	0.942	0.871	0.976	0.853
√	√	√	√	×	0.948	0.873	0.979	0.867
**√**	**√**	**√**	**√**	**√**	**0.954**	**0.874**	**0.983**	**0.881**

The bold row corresponds to the complete ProtT5-MSCRNet framework and is highlighted for visual distinction from the intermediate ablation variants.

**Table 5:** Ablation results of ProtT5-MSCRNet on Test Set 2.

ProtT5	MSC	SE	Aug	Focal	ACC	SN	SP	MCC
√	×	×	×	×	0.952	0.941	0.960	0.901
√	√	×	×	×	0.964	0.956	0.972	0.926
√	√	√	×	×	0.972	0.968	0.978	0.942
√	√	√	√	×	0.978	0.974	0.982	0.955
**√**	**√**	**√**	**√**	**√**	**0.984**	**0.980**	**0.987**	**0.967**

The bold row corresponds to the complete ProtT5-MSCRNet framework and is highlighted for visual distinction from the intermediate ablation variants.

### 2.2 Datasets

To conduct a fair and comprehensive comparison with previously reported anticancer peptide prediction methods, we employed a widely used benchmark dataset originally introduced in earlier studies. The dataset is partitioned into a training subset and two independent evaluation subsets (Bian et al., 2024). Peptide sequences used for model training and the first evaluation set were aggregated from multiple public repositories, including CancerPPD (Tyagi et al., 2015), APD3 (Wang et al., 2016), and SATPdb (Singh et al., 2016). Only peptides with lengths ranging from 5 to 50 residues were considered. To ensure consistency in evolutionary feature availability, sequences that failed to generate valid position-specific scoring matrices (PSSMs) via PSI-BLAST (Shen et al., 2016) were excluded. Sequence redundancy was subsequently reduced using CD-HIT (Fu et al., 2012) by enforcing a 90% sequence identity cutoff, thereby limiting potential bias caused by highly similar peptides. An additional independent test set was introduced to rigorously examine the model’s generalization ability. This dataset was derived from an external source previously reported in the literature and processed using the same filtering and redundancy removal procedures applied to the training data (Agrawal et al., 2021). The final composition of all datasets is summarized in Figure 2.

### 2.3 Evolutionary representation via ProtT5

To overcome the limitations of traditional handcrafted features (e.g., amino acid composition or physicochemical property–based encodings) in capturing complex evolutionary relationships and long-range dependencies among residues, this study employs the ProtT5-XL-UniRef50 model, pre-trained on the large-scale protein sequence database UniRef50, as the underlying feature extractor (Hao and Fan, 2024). This model is built upon the T5 encoder architecture within the Transformer framework and contains approximately 3 billion parameters. Through self-supervised learning on massive unlabeled protein sequences, ProtT5 is capable of capturing deep biological semantics embedded in protein sequences.

During feature extraction, each peptide sequence is fed into the frozen encoder, and the hidden states from the final encoder layer are extracted. In this manner, each amino acid residue in the sequence is mapped to a 1,024-dimensional dense vector. The resulting feature matrix E is defined as:


$$E=\{e_{1},,,e_{2},,,\ldots ,,,e_{L}\},e_{i}\in {\mathbb{R}}^{1024}$$ (1)

where L denotes the sequence length.

Given the relatively small size of the ACP training set and the extremely high dimensionality of the embedding space, the model is prone to overfitting. To mitigate this issue, a feature-space augmentation strategy is introduced during training by injecting small random perturbations ϵ into the embeddings of positive samples to simulate feature variations under different physicochemical environments:


$$\epsilon ∼N(0,0.01^{2})$$ (2)

By applying these perturbations to the original embeddings, the model effectively enlarges the decision boundary of positive samples. This augmentation strategy not only increases data diversity but also encourages the model to learn more robust and intrinsic discriminative features, thereby significantly enhancing its generalization performance on an independent test set.

### 2.4 Multi-scale feature extraction and recalibration

#### 2.4.1 Multi-scale convolutional module

The biological activity of ACPs is closely associated with their secondary structures (e.g., *α*-helices) and local structural motifs (e.g., hydrophobic clusters). Motifs of different lengths often encode biological signals at distinct hierarchical levels. To effectively capture such multi-dimensional structural information, we design a multi-scale convolutional module consisting of three parallel branches. Each branch applies one-dimensional convolutional kernels with sizes k∈{3,,5,,7} to the ProtT5 embedding features, enabling spatial scanning at different receptive field scales.

Specifically, the branch with k=3 focuses on modeling short-range interactions between adjacent residues, such as typical charge alternation patterns. In contrast, the k=5 and k=7 branches are designed to capture longer-range structural units, allowing the model to identify amino acid arrangement patterns spanning approximately two to three helical turns. Each convolutional branch integrates a convolution operation followed by batch normalization (Batch Normalization, BN) and a Rectified Linear Unit (ReLU) activation function. The formulation is given by:


$$X_{k}=\sigma (\mathrm{BN}(\text{Conv}1d_{k}(E)))$$ (3)

where $E\in {\mathbb{R}}^{1024\times L}$ denotes the ProtT5 embedding matrix of the input peptide sequence, L is the sequence length, $\text{Conv}1d_{k}(\cdot )$ represents the one-dimensional convolution operation with kernel size k , BN(·) denotes batch normalization, σ(·) is the ReLU activation function, and $X_{k}$ is the output feature map generated by the branch with kernel size k . Functionally, different kernel sizes are used to capture local sequence patterns and structural motifs at different spatial scales, enabling the model to learn both short-range and relatively longer-range residue interactions. The feature maps generated by the three branches are concatenated along the channel dimension to form a unified multi-scale representation:


$$X_{\text{concat}}\in {\mathbb{R}}^{384\times L}$$ (4)

where $X_{\text{concat}}$ denotes the concatenated multi-scale feature representation, L is the sequence length, and 384 is the total number of output channels obtained by concatenating the feature maps from the three convolution branches. This concatenated representation preserves sequence-wise spatial information while integrating discriminative features extracted from multiple receptive fields.

Through the synergistic integration of multiple receptive fields, this module enables the model to comprehensively characterize ACP structural features across both local and intermediate spatial scales.

#### 2.4.2 Squeeze-and-excitation (SE) block

Given the high dimensionality of the features generated by ProtT5, not all convolutional feature channels contribute equally to ACP discrimination. To identify and enhance the most informative functional channels, such as those strongly associated with hydrophobicity or net charge, we incorporate a Squeeze-and-Excitation (SE) attention mechanism to recalibrate the feature maps.

First, in the Squeeze stage, global average pooling (GAP) is applied along the sequence dimension to compress the spatial information into a channel-wise descriptor:


$$z_{c}=\frac{1}{L}\sum_{i=1}^{L}x_{c}(i)$$ (5)

where $z_{c}$ denotes the channel descriptor of the c -th feature channel after global average pooling, $x_{c}(i)$ represents the activation value at position i in the c -th channel of the input feature map, and L is the sequence length. This operation summarizes the global response of each channel across the entire peptide sequence and provides a compact descriptor for subsequent channel-importance estimation.

Next, during the Excitation stage, two fully connected layers are employed to model nonlinear inter-channel dependencies, followed by a Sigmoid activation function to generate channel-wise importance weights W :


$$W=\text{Sigmoid}(W_{2}\cdot \text{ReLU}(W_{1}\cdot z))$$ (6)

where z is the channel descriptor vector obtained from the squeeze operation, $W_{1}$ and $W_{2}$ are the learnable weight matrices of the two fully connected layers, and W denotes the final channel-wise importance weight vector after sigmoid normalization. Functionally, this excitation operation models nonlinear inter-channel dependencies and assigns larger weights to more informative channels, thereby enhancing biologically relevant feature responses while suppressing less useful or noisy ones.

Finally, in the Scale operation, the learned weights are applied to the original feature map via channel-wise multiplication to achieve feature recalibration:


$$X_{\mathrm{se}}=W⊗X_{\text{concat}}\;$$ (7)

where $X_{\mathrm{se}}$ denotes the recalibrated feature map, W is the learned channel-wise importance weight vector, $X_{\text{concat}}$ is the original multi-scale feature map, and ⊗ denotes channel-wise element-wise multiplication. This scaling operation adaptively reweights each channel according to its relevance to ACP prediction, thereby improving feature selectivity and interpretability.

Through this adaptive reweighting mechanism, the model effectively suppresses redundant or noisy features while emphasizing the most discriminative physicochemical channels, thereby further improving prediction accuracy.

#### 2.4.3 Bi-pooling integration

To alleviate potential information loss caused by dimensionality reduction during pooling, we adopt a bi-pooling integration strategy that combines global average pooling (GAP) (Li and Wang, 2020) and global max pooling (GMP) (Lin and Yang, 2023):


$$V_{\text{pool}}=[\;\mathrm{GAP}(X_{\mathrm{se}})∥\mathrm{GMP}(X_{\mathrm{se}})]$$ (8)

where $V_{\text{pool}}$ denotes the pooled feature vector used for downstream classification, GAP(·) and GMP(·) represent global average pooling and global max pooling, respectively, and ∥ denotes vector concatenation. From a functional perspective, GAP captures the overall distributional trend of sequence features, whereas GMP emphasizes the strongest local activations. Their combination therefore provides a more comprehensive and robust representation for ACP classification.

GAP provides a holistic and stable representation of global sequence characteristics, whereas GMP captures the most salient local functional signals within the sequence. The complementary nature of these two pooling operations enables a more expressive and robust representation for downstream ACP classification.

In addition, to improve the model’s ability to handle hard-to-classify samples, we employ a focal loss–based optimization strategy. The modulating factor ${\left(1p_{i,t}\right)}^{\gamma }$ dynamically down-weights well-classified samples during training, thereby encouraging the model to focus on ambiguous ACP samples whose predicted probabilities lie near the decision boundary. This mechanism effectively enhances the learning of challenging samples and contributes to more stable and discriminative model training.

### 2.5 Sensitivity-oriented loss optimization

In clinical and therapeutic screenings, the cost of false negatives, which refers to misclassifying ACPs as non-ACPs, is typically more detrimental than false positives. A false negative can lead to the omission of a potentially effective therapeutic agent, while false positives can be filtered out in subsequent experimental tests. However, ACP datasets often exhibit class imbalance and overlapping feature distributions, which make it difficult for traditional cross-entropy loss to effectively distinguish between classes. As a result, the model tends to focus on easily classified samples, creating conservative decision boundaries and reducing sensitivity. To address this issue, we utilize Focal Loss (Li et al., 2020), a loss function designed to prioritize hard-to-classify samples during training, improving the model’s ability to handle imbalanced data and enhancing its sensitivity to challenging instances. The feature extraction, feature recalibration, pooling, loss function, and evaluation metrics are defined in Equations 1–13:


$$L_{\text{focal}}=-\sum_{i=1}^{N}\alpha {\left(1-p_{i,t}\right)}^{\gamma }\log(p_{i,t})$$ (9)

where $p_{i,t}$ denotes the predicted probability that sample i belongs to its true class t , α is a weighting factor, and γ controls the strength of hard-sample focusing.

The primary advantage of Focal Loss lies in its hard-sample mining mechanism. The modulating factor ${\left(1p_{i,t}\right)}^{\gamma }$ dynamically suppresses the contribution of well-classified samples with high confidence, while amplifying the influence of ambiguous samples whose predicted probabilities lie near the decision boundary. This mechanism guides the optimization process to focus more on challenging ACP candidates, thereby effectively reducing false negatives and improving sensitivity.

To comprehensively evaluate model performance under class-imbalanced conditions, multiple complementary metrics are employed. Sensitivity (SN) (Sui et al., 2024) measures the model’s ability to correctly identify ACPs and is defined as:


$$\mathrm{SN}=\frac{\mathrm{TP}}{\mathrm{TP}+\mathrm{FN}}$$ (10)

where TP and FN denote true positives and false negatives, respectively.

In contrast, specificity (SP) (Roudsari et al., 2011) quantifies the ability to correctly recognize non-ACP samples:


$$\mathrm{SP}=\frac{\mathrm{TN}}{\mathrm{TN}+\mathrm{FP}}$$ (11)

with TN and FP representing true negatives and false positives.

Overall predictive correctness is measured by accuracy (ACC) (Sui et al., 2025):


$$\mathrm{ACC}=\frac{\mathrm{TP}+\mathrm{TN}}{\mathrm{TP}+\mathrm{TN}+\mathrm{FP}+\mathrm{FN}}$$ (12)

although this metric may be biased in imbalanced datasets.

To provide a more balanced and informative assessment, we further adopt the Matthews Correlation Coefficient (MCC) (Chicco and Jurman, 2020), which jointly considers all four elements of the confusion matrix:


$$\mathrm{MCC}=\frac{\mathrm{TP}\times \mathrm{TN}-\mathrm{FP}\times \mathrm{FN}}{\sqrt{\left(\mathrm{TP}+\mathrm{FP})(\mathrm{TP}+\mathrm{FN})(\mathrm{TN}+\mathrm{FP})(\mathrm{TN}+\mathrm{FN}\right)}}$$ (13)

By integrating a sensitivity-oriented loss function with a comprehensive set of evaluation metrics, the proposed framework prioritizes the identification of true ACPs while maintaining balanced and reliable performance across different classes, which is essential for practical anticancer peptide discovery.

## 3 Results

### 3.1 Comparison with existing methods

To comprehensively evaluate the effectiveness of the proposed ProtT5-MSCRNet, we compared its predictive performance with several representative state-of-the-art ACP prediction methods on two independent benchmark datasets, namely Test Set 1 and Test Set 2. All compared methods were evaluated on the same benchmark datasets and assessed using the same evaluation metrics, ensuring a fair and unbiased comparison. The comparison results are summarized in Tables 1, 2, respectively, using four widely adopted metrics: accuracy (ACC), sensitivity (SN), specificity (SP), and Matthews correlation coefficient (MCC).

As shown in Figure 3, ProtT5-MSCRNet achieves the best overall performance among all compared methods on Test Set 1. Specifically, it attains an ACC of 0.954 and an MCC of 0.881, outperforming the strongest baseline ACP-ESM2 by 2.3 and 5.6%, respectively. These results indicate a substantial improvement in overall prediction reliability and balanced classification performance. In terms of sensitivity, ProtT5-MSCRNet achieves an SN of 0.874, which is comparable to other high-performing models such as ACP-ESM2 and ACP-ML, demonstrating its strong capability to identify true anticancer peptides. More notably, ProtT5-MSCRNet yields the highest specificity (SP = 0.983), indicating an exceptional ability to correctly distinguish non-ACP sequences and effectively suppress false positives. This high specificity, combined with competitive sensitivity, results in a superior MCC value, highlighting the robustness and stability of the proposed model under class-imbalanced conditions. In contrast, earlier deep learning models such as ACP-MHCNN and ACPred-BMF exhibit relatively low specificity and MCC values, suggesting limited discriminative power and less balanced performance. Traditional machine learning–based methods, including ACP-Pred and MACPpred, show improved results but still fall short of the proposed framework, particularly in terms of MCC.

The superiority of ProtT5-MSCRNet is further validated on the more challenging and independent Test Set 2, as reported in Figure 4. On this dataset, ProtT5-MSCRNet achieves an ACC of 0.984, an SN of 0.980, an SP of 0.987, and an MCC of 0.967, outperforming all competing methods by a clear margin across all evaluation metrics.

Compared with ACP-ML, which represents the strongest baseline on Test Set 2, ProtT5-MSCRNet improves ACC and MCC by 5.9 and 12.4%, respectively. The near-perfect balance between sensitivity and specificity demonstrates that the proposed model not only excels at identifying ACPs but also maintains a very low false-positive rate, which is particularly important for practical peptide screening applications.

To complement point-estimate evaluation on relatively small independent test sets, we performed a nonparametric bootstrap analysis to assess the statistical stability of model performance. Specifically, for each independent test set, bootstrap resampling with replacement was conducted for 1,000 iterations while maintaining the original sample size in each replicate. For every bootstrap replicate, ACC, SN, SP, and MCC were recalculated, and the corresponding 95% confidence intervals (95% CIs) were estimated using the percentile method. The bootstrap-based results are summarized in Table 1. On Test Set 1, ProtT5-MSCRNet achieved an ACC of 0.954 (95% CI: 0.935–0.971), an SN of 0.874 (95% CI: 0.817–0.927), an SP of 0.983 (95% CI: 0.969–0.994), and an MCC of 0.881 (95% CI: 0.832–0.924). On Test Set 2, the corresponding values were ACC = 0.984 (95% CI: 0.968–0.996), SN = 0.980 (95% CI: 0.949–1.000), SP = 0.987 (95% CI: 0.969–1.000), and MCC = 0.967 (95% CI: 0.937–0.991). These interval estimates provide additional evidence that the proposed framework remains stable and reliable under limited-sample evaluation settings.

Overall, the consistently strong performance of ProtT5-MSCRNet across both test sets highlights its favorable generalization ability and predictive stability. Taken together, these findings further demonstrate the effectiveness of combining pretrained evolutionary representations, multi-scale convolutional feature extraction, channel-wise recalibration, and sensitivity-oriented optimization for accurate and reliable anticancer peptide prediction.

### 3.2 Effect of different protein embedding methods

To investigate the impact of different protein sequence embedding strategies on the performance of the proposed framework, we conducted a controlled comparative study using multiple pretrained protein language model embeddings. Specifically, ProtT5 embeddings in ProtT5-MSCRNet were replaced with alternative representations, including ESM2 and ProtBERT, while keeping the downstream network architecture, training configuration, and evaluation protocol strictly identical. This design ensures that any observed performance differences can be attributed solely to the embedding methods. The experimental results on Test Set 1 and Test Set 2 are summarized in Tables 2, 3, respectively. Four widely used evaluation metrics were employed, including accuracy (ACC), sensitivity (SN), specificity (SP), and Matthews correlation coefficient (MCC).

As shown in Table 2, the model using ProtT5 embeddings achieves the best overall performance among all embedding variants. In particular, ProtT5-MSCRNet attains an ACC of 0.954 and an MCC of 0.881, outperforming the ESM2-based and ProtBERT-based models by a clear margin. Although the ESM2-based model demonstrates relatively high specificity (SP = 0.955), its sensitivity is noticeably lower, leading to a reduced MCC. This indicates that ESM2 embeddings tend to bias the classifier toward negative samples, resulting in less balanced predictions under class-imbalanced conditions. In contrast, ProtBERT embeddings show inferior performance across all evaluation metrics, especially in terms of MCC, suggesting limited capability in capturing discriminative sequence features for anticancer peptide identification. Overall, ProtT5 embeddings provide the most favorable trade-off between sensitivity and specificity, yielding the highest MCC and the most stable classification performance.

The superiority of ProtT5 embeddings is further confirmed on the more challenging independent Test Set 2, as reported in Table 3. The ProtT5-based model achieves near-optimal performance across all metrics, with an ACC of 0.984, an SN of 0.980, an SP of 0.987, and an MCC of 0.967. Compared with the ESM2-based variant, ProtT5-MSCRNet improves ACC and MCC by 2.6 and 6.5%, respectively, indicating stronger generalization capability and more robust predictive behavior. Although the ESM2-based model remains competitive, its relatively lower MCC suggests suboptimal balance between true positive and true negative predictions. ProtBERT again exhibits the weakest performance, particularly in MCC, highlighting the limitations of earlier protein language models when applied to complex ACP prediction tasks.

### 3.3 Ablation study results

To quantitatively assess the contribution of each component in ProtT5-MSCRNet, we conducted a series of ablation experiments on two independent test sets. The results on Test Set 1 are summarized in Table 4, while those on Test Set 2 are reported in Table 5. The baseline model using only ProtT5 embeddings already shows competitive performance, achieving an ACC/MCC of 0.921/0.812 on Test Set 1 and 0.952/0.901 on Test Set 2, confirming the effectiveness of pretrained evolutionary representations. Introducing the multi-scale convolution (MSC) module consistently improves performance, increasing ACC and MCC to 0.934/0.836 and 0.964/0.926 on the two test sets, respectively, demonstrating the benefit of modeling local structural patterns at multiple receptive fields. Further incorporating the Squeeze-and-Excitation (SE) channel attention mechanism yields additional gains in MCC and specificity, with MCC rising to 0.853 on Test Set 1 and 0.942 on Test Set 2, indicating more effective suppression of redundant features in high-dimensional embeddings. Enabling feature-space perturbation augmentation leads to further improvements in generalization, particularly on Test Set 2, where MCC increases to 0.955, highlighting its role in mitigating overfitting under limited data conditions. Finally, replacing cross-entropy loss with focal loss results in the best overall performance, with the full ProtT5-MSCRNet achieving ACC/SN/SP/MCC values of 0.954/0.874/0.983/0.881 on Test Set 1 and 0.984/0.980/0.987/0.967 on Test Set 2. Overall, the consistent and progressive performance improvements across both datasets confirm that each component contributes positively and that their synergistic integration is essential for accurate, robust, and generalizable anticancer peptide identification.

### 3.4 Model interpretability via t-SNE visualization

To provide an intuitive interpretation of the learned feature representations, we performed t-distributed stochastic neighbor embedding (t-SNE) (Cieslak et al., 2020) on the high-level embeddings extracted from the baseline model and the proposed ProtT5-MSCRNet. The visualization results for Test Set 1 and Test Set 2 are presented in Figure 5. As shown in Figure 5a, the baseline model using ProtT5 embeddings alone exhibits only moderate discriminative ability on Test Set 1. Although the ACP and non-ACP samples show an overall tendency to separate, noticeable overlap remains in the central region, indicating that pretrained embeddings alone are not sufficient to fully capture the task-specific characteristics required for robust ACP identification. By contrast, Figure 5b demonstrates that ProtT5-MSCRNet produces a much clearer separation between the two classes on Test Set 1, with more compact intra-class distributions and reduced inter-class mixing. A similar but more pronounced pattern can be observed on Test Set 2. In Figure 5c, the baseline model already shows a relatively distinct class-wise distribution, suggesting that the learned ProtT5 features possess certain discriminative power on this dataset. Nevertheless, Figure 5d reveals that ProtT5-MSCRNet further improves the feature organization by generating cleaner boundaries and more compact class manifolds, leading to the most distinct separation among all four panels. Overall, the t-SNE visualizations are in good agreement with the quantitative results and ablation analyses. In particular, the proposed ProtT5-MSCRNet consistently yields more discriminative feature representations than the baseline model on both test sets. These results further support the effectiveness of multi-scale feature extraction, channel-wise recalibration, and task-oriented optimization in enhancing ACP-related representation learning. It should be noted that t-SNE is primarily a qualitative visualization tool and does not preserve global distances in the original feature space. These visualization results provide representation-level interpretability evidence by showing how the proposed framework organizes ACP and non-ACP samples into more separable feature manifolds.

### 3.5 Channel-wise comparison with and without SE attention

To further investigate the effect of channel-wise recalibration in ProtT5-MSCRNet, we conducted a comparative channel importance analysis between the baseline model without SE attention and the proposed SE-equipped model. Channel importance was quantified by averaging normalized channel activation strengths across all test samples, and the results are shown in Figure 6. As illustrated in Figure 6a, the baseline model exhibits a nearly uniform channel importance distribution, where most channels contribute similarly to the final prediction. This flat distribution indicates that, without explicit channel-wise attention, the model lacks the ability to selectively emphasize informative channels and thus utilizes high-dimensional feature representations in an indiscriminate manner. In contrast, Figure 6b shows that ProtT5-MSCRNet learns a markedly non-uniform channel importance distribution. A subset of channels receives substantially higher attention weights, while the majority of channels are relatively suppressed, demonstrating that the SE module effectively reweights feature channels according to their relevance for anticancer peptide prediction. To further highlight the most influential channels, we ranked all channels based on their average SE attention scores and visualized the top-20 most important channels, as shown in Figure 6c. These channels exhibit significantly higher normalized importance values than the remaining channels. The corresponding heatmap in Figure 6d provides a complementary visualization, clearly illustrating the gradual decay in channel importance. Overall, this channel-wise comparison provides clear interpretability evidence for the effectiveness of the SE attention mechanism. By transforming an initially uniform channel contribution pattern into a highly selective importance distribution, the SE module enables ProtT5-MSCRNet to focus on the most discriminative feature channels, complementing the quantitative performance gains and t-SNE visualization results. Together, these findings offer channel-level interpretability support for the proposed model by revealing how the SE mechanism selectively enhances discriminative feature channels relevant to ACP prediction.

## 4 Discussion

This study developed ProtT5-MSCRNet, an end-to-end framework for ACP prediction that integrates pretrained evolutionary representations, multi-scale convolutional feature extraction, channel-wise recalibration, and robust optimization strategies. Results on two independent benchmark datasets show that the proposed model achieves consistently strong and well-balanced predictive performance across multiple evaluation metrics, particularly MCC. In addition, the bootstrap-based confidence interval analysis further supports the statistical stability of the framework under relatively limited test-set sizes.

A key finding of this work is that pretrained protein language model representations provide a strong basis for ACP prediction when paired with suitable downstream modeling. Although such embeddings contain rich contextual and evolutionary information, they may also introduce redundancy. Our results indicate that their utility can be better realized through multi-scale local feature extraction and adaptive channel recalibration. This is supported by both the embedding comparison experiments and the ablation study, in which ProtT5-based representations and the associated architectural modules consistently improved performance on independent test sets. The performance advantage of ProtT5-MSCRNet appears to arise from the combined effect of its major components. The multi-scale convolutional module improves the modeling of local sequence patterns and functional motifs, whereas the SE block enhances feature selectivity through channel-wise recalibration. Feature-space perturbation augmentation helps reduce overfitting under limited-data conditions, and focal-loss-based optimization improves sensitivity to difficult ACP samples. Although the use of high-dimensional pretrained embeddings may raise concerns about overfitting, the consistently strong performance on two independent test sets, together with the gains brought by augmentation and focal-loss-based training, suggests that the proposed framework achieves genuine generalization rather than merely memorizing training patterns. Beyond predictive performance, the framework also provides preliminary interpretability support. The t-SNE results show clearer class separation and more compact intra-class distributions than the baseline model, while the channel-wise analysis indicates that the SE mechanism selectively enhances informative feature channels. These findings suggest that the model not only improves ACP prediction accuracy, but also offers insight into how discriminative sequence representations are learned. The main novelty of this study lies in the unified integration of pretrained representations, local multi-scale modeling, channel-wise recalibration, and sensitivity-oriented optimization within a single ACP prediction framework. In this way, the proposed method addresses feature richness, redundancy suppression, generalization ability, and hard-sample learning in a coordinated manner.

Several limitations should also be acknowledged. The current framework relies primarily on sequence-derived information and does not explicitly incorporate other biologically relevant priors, such as physicochemical properties or structural information. In addition, currently available ACP datasets remain limited in size and diversity, which may restrict generalization to highly novel peptide families. Moreover, the interpretability analyses in this work are mainly conducted at the representation and channel levels, while finer-grained biological explanations remain to be explored.

Future work will focus on integrating multimodal biological information, developing more fine-grained and biologically grounded explanation methods, and validating the proposed framework on larger and more diverse external datasets. Given that ProtT5-MSCRNet requires only peptide sequence input and achieves strong predictive performance on independent benchmark datasets, it also has promising potential for practical deployment as a rapid in silico screening tool for ACP identification. In this context, the proposed framework may be further developed into a user-friendly toolbox or web server to support high-throughput peptide screening in early-stage peptide discovery. More broadly, the design principles of ProtT5-MSCRNet may also be extended to other peptide-related prediction tasks in peptide screening and drug discovery.

## 5 Conclusion

In this work, we proposed ProtT5-MSCRNet, an end-to-end framework for ACP prediction that integrates pretrained evolutionary representations, multi-scale convolutional feature modeling, channel-wise recalibration, and robust optimization strategies. Extensive experiments on two independent benchmark datasets demonstrate that ProtT5-MSCRNet consistently outperforms state-of-the-art methods under identical evaluation settings. Specifically, the proposed model achieved ACC/SN/SP/MCC values of 0.954/0.874/0.983/0.881 on Test Set 1 and 0.984/0.980/0.987/0.967 on Test Set 2, indicating strong and well-balanced predictive performance, particularly in terms of MCC. Beyond quantitative evaluation, t-SNE visualization and SE-based channel importance analysis further show that the proposed model learns more discriminative and structured feature representations, while also providing preliminary interpretability evidence from both the feature-space organization and channel-selection perspectives.

Despite these advantages, the current framework still relies mainly on sequence-derived features, which may limit its generalization to highly novel peptide families. In addition, the present interpretability analysis is primarily conducted at the representation and channel levels. Future work will focus on incorporating additional biological priors, such as physicochemical properties and structural information, developing finer-grained explanation methods, and further enhancing the robustness and practical applicability of the proposed framework for peptide screening and related applications.

## Data Availability

The datasets presented in this study can be found in online repositories. The names of the repository/repositories and accession number(s) can be found in the article/Supplementary material.
