# Supplementary material for: ProtT5-MSCRNet: a multi-scale convolutional and channel-recalibrated deep learning framework for anticancer peptide prediction
Source: Front Artif Intell. 2026 Jun 16;9:1856815. doi: 10.3389/frai.2026.1856815 (PMC13315173; doi:10.3389/frai.2026.1856815)
Supplement: Supplementary file 1 [file Table_1.DOCX]

**ProtT5-MSCRNet: A Multi-Scale Convolutional and Channel-Recalibrated Deep Learning Framework for Anticancer Peptide Prediction**

Zhi Li ^1,a^, Zhen Liu ^1,3,a^, Jianguo Zhong ^1,^*, Yuqin Jiang ^2,^*

^1^Department of Rehabilitation Medicine, the Second Affiliated Hospital of Chengdu Medical College (Nuclear Industry 416 Hospital), Chengdu, 610050, China.

^2^The First People’s Hospital of Jiashan, Jiashan Hospital Affiliated of Jiaxing University, Jiashan, 314100, China.

^3^Faculty of Humanities and Social Sciences, Macao Polytechnic University, Macao, 999078, China.

^a^These authors contributed equally

*** Correspondence:**

Jianguo Zhong, Yuqin Jiang

jianguozhong0691@163.com, Jyq13656610126@outlook.com

Table S1. Chronological comparison of representative computational methods for anticancer peptide prediction

| Year | Method | Category | Main strateg | Main characteristics / limitations |
| --- | --- | --- | --- | --- |
| 2019 | ACPred | Traditional machine learning | Handcrafted sequence features with SVM/RF | Early representative ACP predictor; depends heavily on manual feature engineering |
| 2021 | Huang et al. | Two-stage machine learning | Sequential features + physicochemical properties | Supports subtype-related analysis, but still relies on handcrafted descriptors |
| 2021 | ACPredStackL | Ensemble learning | Stacking of multiple ACP predictors | Competitive performance, but framework complexity is relatively high |
| 2021 | ACP-MHCNN | Deep learning | Multi-head CNN with sequence, physicochemical, and evolutionary features | Reduced reliance on manual design, but feature fusion remains relatively shallow |
| 2022 | ACPred-BMF | Deep learning | BiLSTM with attention and multiple feature representations | Improved interpretability, but representation learning capacity is still constrained |
| 2022 | ACP-2DCNN | Deep learning | 2D CNN on dipeptide-based feature maps | Enhances local pattern learning, but representation construction is task-specific |
| 2023 | CACPP | Deep learning | TextCNN with contrastive learning | Learns discriminative sequence features, but does not explicitly exploit protein language model embeddings |
| 2024 | mACPpred 2.0 | Deep learning | Stacked deep learning with integrated spatial and probabilistic features | Strong benchmark performance, but still relies partly on engineered representations |
| 2024 | ACP-ML | Sequence-based model | Sequence-based ACP prediction framework | Strong recent baseline, but does not fully address hierarchical feature recalibration |
| 2024 | iACP-DFSRA | PLM-based deep learning | ProtBert embeddings + dual-channel fusion + attention | Demonstrates benefit of pretrained embeddings, though feature redundancy may remain insufficiently controlled |
| 2025 | ACP-ESM2 | PLM-based deep learning | ESM2 embeddings + CNN | Strong performance with pretrained protein language models, but downstream hierarchical modeling and feature selection can still be improved |
| 2026 | Aegis | Transformer-based deep learning | Transformer-based framework with multi-feature extraction, feature selection, and interpretability analysi | Achieves SOTA performance on independent testing data; however, it still relies on manually designed feature extraction and feature selection procedures rather than end-to-end representation learning |
